# Chemical Characterization and Volatile Profile of Trebbiano di Lugana Wine: A Case Study

**DOI:** 10.3390/foods9070956

**Published:** 2020-07-18

**Authors:** Daniela Fracassetti, Davide Camoni, Lodovico Montresor, Rebecca Bodon, Sara Limbo

**Affiliations:** 1 Department of Food, Environmental and Nutritional Sciences (DeFENS), Università degli Studi di Milano, Via G. Celoria 2, 20133 Milan, Italy; lodovicomontresor@gmail.com (L.M.); rebecca.bodon@unimi.it (R.B.); sara.limbo@unimi.it (S.L.); 2 Enoconsulting srl Centro Servizi e Ricerca Applicata, Via iseo 6/a, 25030 Erbusco (BS), Italy; dcamoni@enoconsulting.it

**Keywords:** Trebbiano di Lugana wine, aromas, aging, sensory analysis, color, storage

## Abstract

In this study, the volatile profile of Trebbiano di Lugana wine was determined and its chemical composition was considered to understand its potential longevity. Seven wine samples produced in different years (2005–2017) were collected by the same winery and analyzed up to 13 years after bottling. Color, total and polymeric phenols, glutathione, free volatiles and sensory characteristics were assessed. The color turned from yellow to an increased brownish hue as the aging time increased; nonetheless, it was stable up to five years from the production. Thirty-six aroma compounds were detected including higher alcohols, esters, and norisoprenoids (β-damascenone and β-oxo-ionone). While higher alcohols did not show a dependence on the different years of production, a decrease of esters was found over aging with the exception of wine produced in 2009, the latter showing higher levels of glutathione that could limit esters’ hydrolysis. The perception of floral and fruity notes was dependent on the storage time with little differences up to five years after bottling. Trebbiano di Lugana wine could be suitable for aging and this aptitude might be further improved also through the proper choice of closure and packaging systems to encourage logistic and marketing strategies.

## 1. Introduction

Trebbiano di Lugana is a white *Vitis vinifera* cultivar mainly cultivated in an Italian area located south near Garda lake (Verona, north of Italy). This grape variety, also known as “Turbiana,” is used for the production of Trebbiano di Lugana wines with recognized appellation of origin (DOC).

Traditionally, the Trebbiano di Lugana grape was considered identical with Trebbiano di Soave and Verdicchio grape varieties, the first one also grown in Verona area, while the second one is diffused in the Marche region (center of Italy). The appellation of Trebbiano di Lugana is reserved to the Verdicchio cultivar cultivated in the Verona area. Nevertheless, the origin of these grape cultivars is still under discussion. Genetic analysis showed that cv Verdicchio and Trebbiano di Soave are identical [1], the latter being similar to Trebbiano di Lugana [2]. However, these three varieties presented distinctive tracts allowing their differentiation to other *V. vinifera* cv. Verdicchio [3]. More recently, the genetic similarity of these three grape varieties, at least for the part of the genome analyzed, was reported by Ghidoni et al. [4], even if traces of three different biotypes of the same variety could remain in relation to phenotypical traits that are environment-dependent (terroir). 

To the best of our knowledge, the volatile profile of Trebbiano di Lugana wine has not been described yet. Among the grape varieties similar to the Trebbiano di Lugana grape, the aroma profile of wine produced with the Verdicchio grape was recently described by Canonico et al. [5]. The authors reported that Verdicchio wine was characterized by fermentative esters such as ethyl butyrate, phenyl acetate, ethyl acetate and isoamyl acetate, the latter being the most abundant. Terpenes, such as linalool, nerol and geraniol, were also found [5]. Recently, the aromatic complexity of Verdicchio wines was described, also relating to its aging. Younger wines were characterized by fruity and tropical notes, while aged wines were distinguished by the presence of norisoprenoids, anise and balsamic notes, related to 3-methyl-2,4-nonanedione and methyl salicylate released by precursors [6]. The presence of varietal thiols, namely 3-mercaptohexan-1-ol and its acetate form, conferring tropical and citrus notes has been documented in Trebbiano di Lugana wine. Winemaking strategies allowing for the protection and maintenance of these desired aromas were also described [7]. Moreover, the abundance of glutathione, a natural antioxidant, resulted in particular interest for reducing the use of exogenous antioxidants, i.e., sulfur dioxide [7], and because of its important role in limiting the loss of varietal thiols [8].

This research aimed at conducting a preliminary evaluation on some quality traits of Trebbiano di Lugana produced in different years, between 2005 and 2017, and stored in dark bottles up to 13 years from the respective winery. The potential longevity of Trebbiano di Lugana wine was considered by trying to identify those chemical, volatile and sensorial markers affected by the storage timing period, although different vintages were considered. Volatile compounds were assessed by Solid Phase Microextraction (SPME) technique coupled with gas chromatography-mass spectrometry (GC-MS) and the perceived aromatic notes were described. The ultimate goal is the presentation and discussion of a case study to understand which quality attributes, among those considered, could be used in a more structured aging trial since Trebbiano di Lugana has not been studied so far. The parameters taken into account in the study were color, phenolic components, volatile compounds and sensory characteristics, as they could be majorly affected by the storage time.

## 2. Materials and Methods 

### 2.1. Wine Samples

Seven wine samples were collected in a winery and they were produced with Trebbiano di Lugana grapes in seven different years—2005, 2009, 2011, 2013, 2015, 2016 and 2017—that were analyzed in 2018, thus 13, 9, 7, 5, 3, 2 and 1 year after bottling, respectively. Comparable winemaking procedures were applied over the production period investigated. The grape was harvested based on its technological maturity and its selection was also carried out prior to the winemaking in order to use the grape with the best quality. The winemaking process was carried out under reductive conditions; the pressing was carried out in an inert environment with the addition of sodium metabisulfite (100 mg/L). The free and first-run juice underwent static sedimentation with pectolytic enzyme addition. Once the alcoholic fermentation (conducted at a controlled temperature between 14 °C and 16 °C) was completed, sodium metabisulfite was added (100 mg/L) and the wine was kept in a stainless-steel tank for about six months. After tartaric and protein stabilization and clarification, the wine was added with sodium metabisulfite (100 mg/L), bottled under nitrogen insufflation in green glass bottles and closed with agglomerated cork stoppers (DIAM type). All the bottles’ necks were covered with an aluminium foil to limit the oxygen transfer at the interface and through the cork. All the bottles were stored horizontally in the winery cellar under controlled conditions of temperature and humidity and were protected from light.

### 2.2. Determination of Chemical Parameters 

The chemical parameters were determined by Enoconsulting (Erbusco, BS, Italy), a UNI CEI EN ISO/IEC 17025-accredited laboratory. The parameters investigated were total phenols (spectrophotometric method, Rev 01 11/03/05) [9], polymeric phenols (spectrophotometric method based on *p*-(dimethylamino) cinnamaldehyde) [10], iron [11] and copper [12]. The absorbance readings were carried out and wavelengths 280 nm and 320 nm were determined, after proper dilution of wine samples with water, for the total phenol index (TPI) and total hydroxycinnamic acid index [10,13].

The color parameters were evaluated according to the CIELab analysis [14]. The values of the Chroma (Equation (1)), hue angle (Equation (2)) and ΔE (difference between two colors) (Equation (3)) were also calculated.

(1) $$Chroma=\sqrt{a^{*}{}^{2}\times b^{*}{}^{2}}$$

(2) $$Hue\;angle=arctg\;\frac{\;b^{*}}{\;a^{*}}$$

(3) $$\Delta E=\sqrt{{\left(L^{*}{}_{2017}-L^{*}{}_{YEAR}\right)}^{2}+{\left(a^{*}{}_{2017}-a^{*}{}_{YEAR}\right)}^{2}+{\left(b^{*}{}_{2017}-b^{*}{}_{YEAR}\right)}^{2}}$$

The calculation of each ΔE value was done by comparing the color attributes related to the youngest wine sample (one year old) and those of the wine samples produced in the previous vintages considered (“YEAR” of production). 

The ΔE values were considered by means of the classifications following those reported in [15]:

- ΔE < 0.2: Color difference not noticeable;
- 0.2 < ΔE < 0.5: Color difference is very little;
- 0.5 < ΔE <1.5: Color difference is little;
- 2 < ΔE < 3: Color difference noticeable;
- 3 < ΔE < 6: Color difference easily noticeable;
- 6 < ΔE < 12: Color difference strongly noticeable;
- ΔE > 12: Different colors.

Beside the color analysis by CIELab, spectrophotometric readings at 420 nm were carried out as a marker of yellow color.

Each analysis was conducted in duplicate from two bottles.

### 2.3. Evaluation of Aromatic Profile

Free volatile compounds were determined through Solid Phase Micro Extraction (SMPE)/GC-MS. The fiber was a carboxen-polydimethylsiloxane-divinylbenzene (CARPDMS-DVB; 50/30 μm × 1 cm) (Supelco, Bellefonte, PA, USA). The SPME was carried out with an autosampler (HTA autosampler, Brescia, Italy) set at the following conditions: incubation for 5 min at 40 °C; agitation 10-s on and 3-s off; extraction for 30 min; desorption for 20 min. The GC/MS equipment was a Perkin Elmer Autosystem XL Gas Chromatograph coupled with a Turbomass Mass Spectrometer (Perkin Elmer, Italy). The separation was achieved by a Stabilwax-MS column (30 m × 0.250 mm × 0.25 μm) (Restek, Bellefonte, PA, USA) using helium as a carrier gas at 1 mL/min flow rate. The oven temperature was initially set at 40 °C and held for 5 min, ramped at 1.5 °C/min up to 220 °C and held for 10 min. The transfer line temperature was set at 230 °C and the source temperature was set at 250 °C. The mass spectrometer operated in electron ionization mode at 70 eV using the full scan mode. The MS detector registered the *m/z* in the range from 33 Da up to 350 Da. The ions used for identification of target molecules were chosen according to the National Institute of Standards and Technology (NIST) MS Search 2.0 library fixing a fitting value (R) of minimum 90%, validated by external comparisons of ion fragmentation patterns and by calculating the linear retention index (LRI) according to Van der Dool and Kratz [16] when running an alkane standard solution (C8–C20, Merck, Milan, Italy). The wine sample (2.5 mL) was diluted 1:1 with water (2.5 mL) in a 20 mL SPME vial where 1.45 g of sodium chloride was present and added with 2-methyl-1-penthanol as internal standard (IS) at a concentration of 2.5 mg/L from a stock solution in 10% ethanol (*v*/*v*) of. The vial was tightly closed prior to the analysis. Each analysis was conducted in duplicate from two bottles.

Results were expressed as relative concentrations (µg/L or mg/L) referred to IS. A Five-point calibration curve was obtained for the IS (1 mg/L to 15 mg/L) using the wine sample produced in 2017 as a matrix.

### 2.4. Sensory Analysis

The sensory analysis was carried out by a panel composed of nine expert judges (three female, six male, average age of 40). The session consisted firstly of the definition of the descriptors which were chosen according to consensus method [17] through the tasting of wines produced in 2005, 2013 and 2017. Once the descriptors were selected, the judges were calibrated by tasting the wine sample produced in 2017 that was used as reference considering the median values assigned by the panel for each descriptor selected. For the wine sample tasting, a nine-point scale was used, with 1 meaning “not perceived” and 9 meaning “extremely perceived”. The quantitative profile was performed for all the wines presented in a randomized order including a replicate of a wine sample (produced in 2015) in order to evaluate the replicability of the judges. 

The discriminant capacity (*D*) was calculated as follows:

(4) $$D=\frac{Standard\;deviation\;\left(d\right)}{Standard\;deviation\;\left(p\right)}\times 100$$

where *Standard deviation* (*d*) corresponded to the standard deviation of the scores assigned by the judge for each descriptor, and *Standard deviation* (*p*) corresponded to the standard deviation of the panel.

The replicability (*R*) was calculated as follows:

(5) $$R=\frac{\left|x1-x2\right|}{y}\times 100$$

where *x*1 and *x*2 were the two scores assigned to the wine sample replicated and *y* was the deviation of scores assigned [18,19].

The discriminant capacity of the judges was set at 20% and the replicability was set at 75%.

Selected descriptors belonged to the visual, olfactory, taste-tactile and retro-olfactory sensations that are listed as follows:

- Visual descriptors: yellow intensity, clarity;
- Olfactory descriptors (O): intensity, fruity, boxwood/grapefruit/passion fruit, floral, oxidized/marsala, honey;
- Taste-tactile descriptors: body, acidity, alcohol/heat, savory/salty, persistence, bitter;
- Aftertaste descriptors (AF): intensity, persistence, fruity, peach, marsala/honey/oxidized, green.

### 2.5. Statistical Analysis

The statistical analysis was performed with SPSS Win 12.0 program (SPSS Inc., Chicago, IL, USA). One-way ANOVA was carried out to determine the significant differences related to chemical parameters, volatile compounds and sensory analysis. Post-hoc Fischer LSD (α = 0.05) was carried out for the variables showing a significant storage time effect. Correlation indexes were determined among the chemical parameters and volatile compounds through the Pearson Correlation considering the critical value of 0.755 (*df* = 5, α = 0.05). The principal component analysis (PCA) was performed with Statistica 12 software (Statsoft Inc., Tulsa, 269 OK, USA) on auto-scaled data for an overall overview of the different wines considering the chemical parameters, the aroma compounds showing storage-dependent change (decanoic acid, 1-octanol, 1-decanol, furfural, ethyl acetate, ethyl butanoate, ethyl isovalerate, isoamyl acetate, ethyl hexanoate, hexyl acetate, ethyl decanoate, dimethyl succinate, ethylphenyl acetate, phenylethyl acetate, ethyl dodecanoate, β-damascenone, linalool) and significant sensory descriptors (yellow intensity, fruity_O, boxwood/grapefruit/passion_O, floral_O, oxidized/marsala_O, honey_O, acidity, fruity_AT, peach_AT, marsala/honey/oxidized_AT). The regression between the storage time and the PC1 values was carried out.

## 3. Results

The potential longevity and the attitude to aging of Trebbiano di Lugana wine was evaluated by considering seven wines stored from 1 to 13 years but produced in different years between 2005 and 2017 and stored in dark bottles by the same winery.

Particular attention was given to color, phenolic components, volatile compounds and sensory characteristics. Moreover, to the best of our knowledge, the volatile profile of Trebbiano di Lugana wine was determined for the first time.

### 3.1. Chemical Parameters 

An evident color change was found due to the increased time of storage. Indeed, the younger wines (produced in 2015–2016) showed L* values (lightness) higher than 99, while for the 2005 vintage the L* value was the lowest, also showing the highest value of chroma (Table 1). Both L* and chroma were highly correlated to the storage time, negatively and positively for L* (−0.96) and chroma (0.98), respectively (Table S1). This difference in color attributes can be related to the oxidative browning that led to an increase of color intensity and a decrease of lightness values [20,21]. Furthermore, the color change can be evaluated by observing b* and a* whose values significantly increased with increasing storage time. In general, a global increase of a* (towards red) and b* (towards yellow) is associated to a color change from pale yellow to yellow-amber. In particular, b* results positively correlated to storage time (0.98) (Table S1). To compare the variation of color over time, color differences (ΔE) were determined. No difference was found between wines stored for a shorter time (produced in 2015–2017, respectively) as ΔE was lower than 1 (Table 1), indicating that the color difference is very small [15]. The comparison among wine produced in 2017 (one year old) and those from 2011, 2009 and 2005 (7, 9 and 13 years old) showed a color difference that was easily noticeable for the 2011 vintage (7-years old), strongly noticeable for the 2009 (nine years old) and a different color for the 2005 (13 years old). It is interesting to note that even after five years from the production the color difference is only noticeable, suggesting the possibility that wine lends itself to aging. 

In addition to the color analysis by CIELab, spectrophotometric readings were performed, considering the absorbance values at 420 nm [22]. Similar to the CIELab data, these values significantly increased as the storage time increased due to the browning of the wine caused by the oxidation of the phenolic compounds [22,23]. The absorbance values at 420 nm did not show any significant differences between wines produced in 2017, 2016 and 2015 (Table 1). For wines with a longer storage, the absorbance values at 420 nm increased quickly up until the wine produced in 2005 (0.303). In particular, the absorbance values at 420 nm found for wine from 2013 (five years old) (0.109) was almost double compared to that of 2015 (three years old) (0.063). These data suggest that during the first three years of storage, the color stability of wine could be related to the presence of antioxidants such as glutathione, preventing the oxidation phenomena and browning [8]. In particular, the latter antioxidant results were negatively correlated with a*, b* and absorbance values at 420 nm (Table S1). The decrease of sulfur dioxide could be also expected since this antioxidant correlates with the increase of absorbance at 420 nm [24].

A relation between absorbance values at 280 nm (total phenols) and 320 nm (hydroxycinnamic acids) related to the storage time was not found. The influence of vintage could be stronger than the one related to the storage time. Similarly, total and polymerized phenols did not show a storage time-dependent trend. As well, the ratio of total phenols/polymerized phenols indicated a major role played by the vintage instead of the storage time (Table 2). 

Transition metals, such as iron and copper, are usually found in wine and their presence is due to soil, vineyard treatments and winemaking tools [25]. The levels of transition metals ranged between 0-0.5 mg/L for iron [25] and 0.1–0.3 mg/L for copper [22]. Iron and copper are catalysts for oxidation reactions and can participate in oxidative phenomena, such as oxidative browning, even at low concentrations [25,26], as well as causing iron and copper casse. In order to limit these alterations, their contents should be less than 5 mg/L for iron and 0.3–0.5 mg/L for copper [25,27]. Iron and copper contents in the wines analyzed were lower than the values considered responsible for wine instability (Table 2). However, for the wines with longer storage, the content of iron was slightly higher suggesting an influence time-dependent as showed by its positive correlation with the storage time (0.74) (Table S1).

**Figure 1:**
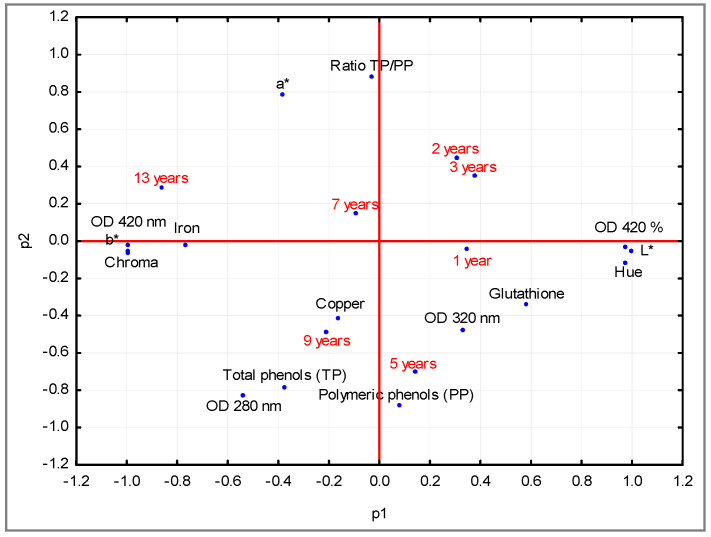
Bi-plot (p1 vs. p2) obtained for chemical parameters of the investigated Trebbiano di Lugana wines (1–13 years of storage).

A further confirmation was obtained by the principal component analysis (PCA) (Figure 1). Two components explained 64% of the total variance, 40% and 24% for P1 and P2, respectively. The values of b*, chroma, absorbance at 420 nm and the content of iron were the chemical parameters affecting Trebbiano di Lugana wine produced in 2005. On the opposite side, the values of L*, hue and absorbance percentage at 420 nm were more related to the younger wines. PCA showed the distribution of wine samples related to storage time (p1), with the chemical parameters that were majorly affected by the storage time being L*, b*, chroma, hue, absorbance values at 380 nm and 420 nm and iron. The phenol indexes were more influenced by the year of production instead of storage time. 

### 3.2. Aroma Profile

The aroma profile was determined for the wines under study by SPME-GC/MS and 36 compounds were detected (Table 3). The aroma profile of the wine produced with Verdicchio grapes [5] and the aromatic profile of the wine produced in 2017 with Trebbiano di Lugana grapes showed some differences. Even if both wines were mainly characterized by esters of a fermentative origin, less esters and higher alcohols were reported by Canonico and co-authors [5] in the Verdicchio wine. Such a differences can be also related to the fermenting yeast [28]. Seventeen esters and nine higher alcohols (out of the 36 volatile compounds identified) were detected in Trebbiano di Lugana wine produced in 2017. In Verdicchio wine, three different monoterpenes were identified, including linalool, nerol and geraniol [5,29], while in Trebbiano di Lugana wine only linalool was detected. Trebbiano di Lugana wine also presented two norisoprenoids, β-damascenone and 3-oxo-β-ionone; the first one was also found in Verdicchio wine [29].

#### 3.2.1. Fatty Acids

Medium- and long-chain fatty acids are produced during alcoholic fermentation by yeasts through fatty acid pathways with acetyl-CoA as the key substrate [40]. Acetic acid and three medium-chain fatty acids (C6, C8, C10), including hexanoic acid, octanoic acid and decanoic acid, were detected in all the analyzed wines. These four fatty acids represent 12% to 16% of the total volatile compound content. Their concentration did not seem to be affected by prolonged bottle storage, except for decanoic acid whose amount was higher in wines produced in 2016 and 2017 (2.14 mg/L and 2.17 mg/L, respectively) and decreased with storage. The content of acetic acid was less than 75 μg/L in all the wine samples.

#### 3.2.2. Alcohols

Higher alcohols are produced by the metabolism of yeasts during alcoholic fermentation starting from carbohydrates or amino acids through the Ehrlich reaction [41]. Nine higher alcohols were detected, including isoamyl alcohol, 1-hexanol, 1-octanol, 2-ethyl-1-hexanol and 2-phenylethanol. Their content in the different wines ranged from 13% to 28% of the total volatile compounds identified. As observed for acids, there was no decrease in their concentration with storage time, except for decanol.

#### 3.2.3. Aldehydes

The two aldehydes detected were furfural and benzaldehyde. The presence of furan compounds in wines leads to sensory changes, including the color and aroma of wines [42]. 2-furfural, 5-methyl-2-furfural and 5-hydroxymethyl-2-furfural derive from sugars and they have been strongly correlated with the presence of sotolon in aged wines [43]. In addition, the above-mentioned furan compounds are considered aging markers of oxidized wines, such as the Madeira ones [43,44]. In wines produced under the oxidative winemaking process, increased concentrations of these aromas have been reported following five year aging in barriques [43,45]. Furfural is also present in passito wines, such as Caluso passito DOC; the origin of furan compounds in passito wine has been associated with the degradation of residual sugars occurring during long aging times [46]. Furthermore, furfural was also detected in Champagne wine after two years of aging in a bottle [47] and in young wines produced with Cabernet Sauvignon and Chardonnay grapes [48]. Furfural was detected in the wine stored longer than three years; however, its influence on the sensory profile of white wine might be negligible due to its high perception threshold (14 mg/L) [35]. This furan compound showed a constant increase during storage, reaching the highest levels in wine with the longest storage (13 years) (39.04 ± 6.17 μg/L), therefore potentially more oxidized wines. A further evidence that this compound can be considered a marker of storage was obtained by its positive correlation with storage time (0.87) (Table S2). Benzaldehyde is characterized by a lower perception threshold in comparison to furfural (5 μg/L) and it can confer notes of sugar and almond [42]. This compound was detected only in the youngest wines in concentrations of 45.08 ± 1.53 μg/L and 49.65 ± 4.55 μg/L, respectively, for wine produced in 2017 and 2016.

#### 3.2.4. Esters

Fermentative esters are the aromatic compounds conferring fruity and floral notes and they are involved into the aromatic finesse of young white wines produced with neutral grapes [49]. Eighteen esters were found in the wines analyzed, eight of them were ethyl esters of fatty acids and four belonged to the acetate form of higher alcohols that can derive from the amino acid metabolism of yeasts [50]. The contents of ethyl ester and amyl ester showed a decreasing tendency during storage that can be associated with the loss of fruity and floral notes of white wines during bottle storage [43]. The esters were the most abundant class of aromatic compounds, representing more than 60% of the total for all the vintages, with a maximum of 74% for the youngest wine (2017) (34.93 ± 0.70 mg/L). Decreased content of esters was found between wines stored for one year and two years (−28%) (Table 3). Lower content of total esters was observed for the increasing time of bottle storage in accordance with the expected degradation of these volatile compounds during aging due to acid hydrolysis [42], except for the wine produced in 2009. The latter wine contained a higher content of glutathione (Table 2) that could exert a protective effect against esters’ decay [51,52]. The protection of certain esters against degradation during the storage is related to the redox and nucleophilic properties of GSH thanks to its the free sulfhydryl moiety [53]. The acetic esters, including isoamyl acetate, acetic acid, hexyl ester and phenylethyl acetate, significantly decreased from the wine stored for one year to that stored for three years. A similar trend was observed for other ethyl esters, including ethyl decanoate acid and ethyl dodecanoate, while ethyl hexanoate and ethyl lactate remained unchanged during storage. In particular, significant correlations were found between storage time and ethyl isovalerate, dimethyl succinate, phenylethyl acetate and diethyl malate (Table S2).

#### 3.2.5. Norisoprenoids

Norisoprenoids (C13) derive from the degradation of carotenoids (C40). The main norisoprenoids are represented by β-damascenone, which gives floral notes and exotic fruit and apple jam notes, and β-ionone has floral notes of violet. β-damascenone and 3-oxo-β-ionone were detected. The first one was detected in highly variable concentrations between different wines submitted to different storage times and its decrease could be related to bottle storage, with the exception of the 2015 wine in which it was not detected. 3-Oxo-β-ionone was only found in the youngest wine (2017) and it highly correlated with storage time (0.74).

#### 3.2.6. Monoterpenes

Linalool was the only monoterpene detected in Trebbiano di Lugana wine samples. Except for the wine sample produced in 2015 in which linalool was not detected, lower levels were found as the storage time increased, apart from wine of 2009. This could be ascribed to the high content of glutathione (Table 2). Besides the protection of certain esters, glutathione can limit the oxidation of terpene alcohols into the respective terpene oxide [53].

### 3.3. Sensory Evaluation

Among the descriptors selected by the panelists, significant differences were found for yellow intensity, olfactory (O-) intensity, O-fruity, O-grapefruit/boxwood/passion fruit, O-floral, O-oxidized/marsala, O-honey, acidity, aftertaste (AF-) fruity, AF-peach and AF-marsala/honey/oxidized (Table 4). 

Concerning the yellow intensity descriptor, significant differences were found for the older samples, namely 2005, 2009 and 2011. The highest score (8/9) was assigned to the wine of 2005, while there were no differences in terms of intensity of the yellow color between the wines produced in 2011 and 2009 (score: 6/9). This result is supported by both the color analysis (Table 1) and the absorbance values at 420 nm (Table 2). In fact, depending on the storage time, increased values of absorbance at 420 nm, chroma and a decreased value of hue were reported [23,54]. The yellow intensity descriptor did not result in a significant difference between the wines from 2017, 2016, 2015 and 2013 for which the panel assigned the same score (5/9), as supported by CIELab analysis and ΔE values (Table 1).

**Figure 2:**
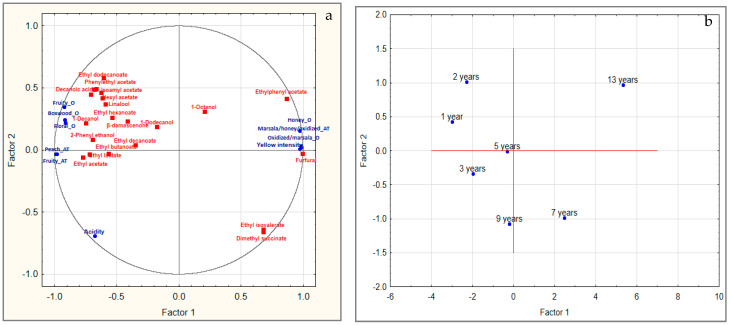
Projection of the (**a**) scores and (**b**) loading on the factor-plane obtained for aroma compounds and sensory scores of the investigated Trebbiano di Lugana wines (1–13 years of storage). The variables considered in the PCA showed significant differences among wine samples. The sensory variables (in blue) were set as active variables and volatile variables (in red) as supplementary variables. Legend: O, olfactory; AT, after taste.

Considering the olfactory attributes, the lowest O-intensity was found for the wines produced in 2009, 2013 and 2015. In order to better clarify the relation between sensory attributes and volatile compounds, PCA was carried out, taking into account the effect of the storage time (Figure 2). The results showed that the first two components were significant in explaining 95% of the total variance, of which 88% was explained by P1 and 7% by P2. The wine samples were distributed on P1 as a function of the time of storage, and the youngest ones were mainly characterized by the presence of esters. As storage time increased, the wines stored for 13 and seven years (2005 and 2011, respectively) were characterized by intensity of yellow color, honey, marsala and oxidized descriptors associated with furfural (Figure 2). P2 was related to the vintage whose role will be further investigated in future studies. The decreased perception of fruity note for increasing storage time (Figure 2) can be associated with the decay of ester content (Table 3). The esters described with fruity notes and associated with the perception of the fruity descriptor were ethyl acetate, ethyl butanoate, isoamyl acetate, ethyl hexanoate, hexyl acetate, ethyl lactate and ethyl dodecanoate; β-damascenone was also included (Figure 2). Similarly, the perception of floral notes decreased for longer storage as the contents of higher alcohols (1-hexanol, 1-octanol, 2-ethyl-1-hexanol, 2-phenylethanol), esters (ethyl butanoate, ethyl isovalerate, ethyl decanoate, acetic acid, phenylethyl acetate, ethyl dodecanoate), norisoprenoids (β-damascenone) were significantly lower (Table 3). Interestingly, the wines produced in 2009 and in 2015 showed the same score for floral descriptors (4/9) (Figure 2). This finding was unexpected and is difficult to explain; the higher content of glutathione detected in the 2009 wine could limit the loss of aroma compounds, including esters and monoterpenes [51,52,53]; particularly, favorable vintage or limited oxidative phenomena could preserve the fruity notes and, consequently, the floral and fruity notes. Moreover, increased scores were found for oxidized/marsala descriptors associated with the wine storage, with the exception of the wine from 2009 (Table 4). Likewise, the honey descriptor, a possible sensory marker of oxidative reactions, was perceived more in the 2005 and 2011 wines than the other samples (Figure 2). For the olfactory perception of boxwood/grapefruit/passion fruit descriptors, three groups were identified as wines produced in 2005, 2009 and 2011, wines of 2013 and 2015 and wines of 2016 and 2017 (Figure 2). Similar to the results related to the fruity and floral notes, the boxwood/grapefruit/passion fruit attribute better described the younger wines (2016 and 2017) (Figure 2) and its perception was dependent on the storage time, maybe due to the degradation of varietal thiols occurring during aging [55].

The perception of selected aftertaste descriptors showed the same trend found for the olfactory ones. For fruity and peach descriptors, the scores assigned by the judges decreased as storage time decreased (Table 4). These sensory attributes were mainly associated with wines produced in 2016 and 2017 and to esters, including ethyl acetate, ethyl butanoate, isoamyl acetate, ethyl hexanoate, hexyl acetate, ethyl lactate and ethyl dodecanoate, and β-damascenone (Table 3, Figure 2). The marsala/honey/oxidized descriptor showed significantly higher scores for wines produced in 2005 and 2011, while those produced in 2009, 2013, 2015 and 2017 were not significantly different (Table 4). As the PCA showed, the oldest wines were described by the presence of furfural (Figure 2), a compound described as a marker of aging [43,44]. It appeared evident that wine from 2011 was more susceptible to the phenomena of oxidative aging than the wines from 2009 and 2013, as furfural concentrations increased even if they were lower than their perception threshold. In the 2009 wine, the oxidative phenomena were less evident compared to that produced in 2011, also supported by lower furfural content compared to the wine produced in 2013 (Table 3).

The PC1 values were considered as a function of storage time, as proposed by other authors [56,57]. A time-dependent linear distribution of the wine samples was found, excluding the wine produced in 2009 that deviates from this trend (Figure S1). This further indicates the strong dependence on storage time of certain volatile and sensory parameters that can be considered as a marker of storage.

## 4. Conclusions

The volatile profile of wines produced with the Trebbiano di Lugana grape was first described by considering wines stored up to 13 years and trying to select quality attributes that were more sensitive to time despite the intrinsic variability of the original samples. Storage time-dependent markers were identified by considering the overall composition and the sensory analysis of Trebbiano di Lugana wine. Among them, a furan derivative, the furfural, was identified in wines stored for more than three years. Browning phenomenon occurred with noticeable color changes in wines store for a period longer than five years. Certain markers were more sensitive to the storage time (i.e., fruity notes) and their monitoring can be suitable for a shelf life study with particular attention on Trebbiano di Lugana wine, but also potentially applicable for shelf life studies related to white wine produced with other grape varieties. The differentiation between young and old wines was achieved, the latter being appreciated even if a decrease of fruity and floral notes was observed for storage longer than three years; nonetheless, this decrease was little until five years after bottling. The possibility of producing long-aging Trebbiano di Lugana wine could be applicable by considering appropriate packaging, effective in the maintenance of its chemical and sensory characteristics. Further researches will also concern the evolution of this wine, taking into account different types of closure that can play an important role in preserving certain fruity and tropical notes for longer, as well as the effect of the vintage on production. Moreover, the methods presented can be applicable to study the evolution of other white wines to better understand which quality attributes are more sensitive to the time of storage.

## Figures and Tables

**Table 1:** Color parameters determined for the investigated Trebbiano di Lugana wines.

Parameter	LS #	Years of Storage						
		13 (2005)	9 (2009)	7 (2011)	5 (2013)	3 (2015)	2 (2016)	1 (2017)
L*	ns	94.14 ± 2.82 ^a^	97.24 ± 2.92 ^a^	97.54 ± 2.93 ^a^	98.51 ± 2.96 ^a^	99.26 ± 2.97 ^a^	99.16 ± 0.97 ^a^	99.18 ± 0.98 ^a^
a*	**	−0.38 ± 0.01 ^a^	−1.37 ± 0.04 ^b^	−1.08 ± 0.03 ^c^	−1.22 ± 0.01 ^d^	−0.84 ± 0.03 ^e^	−0.71 ± 0.02 ^f^	−1.02 ± 0.03 ^g^
b*	**	19.16 ± 0.36 ^a^	11.87 ± 0.32 ^b^	10.73 ± 0.24 ^c^	7.84 ± 0.14 ^d^	4.56 ± 0.14 ^e^	4.48 ± 0.16 ^e^	5.18 ± 0.16 ^f^
Chroma	**	19.16 ^a^	11.95 ^b^	10.78 ^c^	7.94 ^d^	4.64 ^e^	4.54 ^e^	5.28 ^f^
Hue	*	91.16 ^a^	96.61 ^b^	95.76 ^b^	98.83 ^c^	100.4 ^d^	99 ^cd^	101.12 ^d^
ΔE		14.87	6.97	5.78	2.75	0.65	0.76	--
OD 420 nm (AU)	**	0.303 ± 0.01 ^a^	0.178 ± 0.01 ^b^	0.155 ± 0.00 ^c^	0.109 ± 0.00 ^d^	0.063 ± 0.00 ^e^	0.064 ± 0.00 ^e^	0.071 ± 0.00 ^e^
OD 420 (%)	**	72.7 ^a^	78.1 ^b^	77.5 ^c^	80.7 ^d^	82.9 ^e^	82.1 ^e^	84.5 ^e^

Years of production are reported in brackets. Legend: OD, optical density; AU, absorbance unit. ΔE compared the color difference with wine sample of vintage 2017. Different letters mean significant differences (F-test, *p* < 0.05). #: LS, Level of Significance: ns, non-significant; *, *p* < 0.01; **, *p* < 0.001.

**Table 2:** Chemical parameters determined for the investigated Trebbiano di Lugana wines.

Parameter	LS #	Years of Storage						
		13 (2005)	9 (2009)	7 (2011)	5 (2013)	3 (2015)	2 (2016)	1 (2017)
Total Phenols (TP; mg/L)	**	201 ± 6 ^a^	208 ± 6 ^a^	197 ± 6 ^a^	216 ± 5 ^b^	165 ± 5 ^c^	183 ± 6 ^d^	204 ± 6 ^a^
Polymeric Phenols (PP; mg/L)	**	5.1 ± 0.1 ^a^	7.5 ± 0.1 ^b^	4.4 ± 0.1 ^c^	15.7 ± 0.4 ^d^	4.6 ± 0.1 ^c^	3.3 ± 0.0 ^e^	6.7 ± 0.1 ^f^
Ratio *TP*/*PP*	**	39.41 ^a^	27.73 ^b^	44.7 ^c^	13.76 ^d^	35.87 ^e^	55.45 ^f^	30.45 ^g^
OD 280 nm (AU)	**	8.90 ± 0.29 ^a^	9.49 ± 0.31 ^b^	8.41 ± 0.29 ^c^	9.58 ± 0.27 ^b^	7.14 ± 0.25 ^d^	7.35 ± 0.24 ^d^	8.14 ± 0.24 ^c^
OD 320 nm (AU)	**	4.85 ± 0.15 ^a^	5.81 ± 0.17 ^b^	5.36 ± 0.16 ^c^	6.52 ± 0.16 ^d^	6.23 ± 0.19 ^d^	5.35 ± 0.15 ^c^	4.69 ± 0.16 ^a^
Glutathione (mg/L)	**	4 ± 0.2 ^a^	11 ± 0.4 ^b^	7 ± 0.1 ^c^	9 ± 0.2 ^d^	6 ± 0.1 ^c^	11 ± 0.2 ^b^	14 ± 0.4 ^b^
Iron (mg/L)	**	0.96 ± 0.05 ^a^	0.84 ± 0.02 ^b^	0.22 ± 0.03 ^c^	0.30 ± 0.02 ^d^	0.35 ± 0.04 ^d^	0.42 ± 0.05 ^e^	0.42 ± 0.03 ^e^
Copper (mg/L)	*	<0.05 ^a^	0.11 ± 0.01 ^b^	<0.05 ^a^	<0.05 ^a^	<0.05 ^a^	0.06 ^c^	<0.05 ^a^

Year of production is reported in brackets. Legend: OD, optical density; AU, absorbance unit. Different letters mean significant differences (F-test, *p* < 0.05). #: LS, Level of Significance: ns, non-significant; *, *p* < 0.01; **, *p* < 0.001.

**Table 3:** Aroma profile for Trebbiano di Lugana wines. Concentration (means ± standard deviations) is expressed in μg/L except ^§^ in mg/L as equivalent to 2-methyl-1-penthanol.

Compound	LS #	Perception Threshold	LRI	Descriptors	Years of Storage						
					13 (2005)	9 (2009)	7 (2011)	5 (2013)	3 (2015)	2 (2016)	1 (2017)
*Acids*											
Acetic acid	***	200,000 [30]	1455	Acid, unpleasant	43.93 ± 8.94 ^a^	48.79 ±2.63 ^a^	56.07 ± 2.07 ^b^	48.33 ± 5.96 ^a^	48.74 ± 5.73 ^a^	53.59 ± 1.58 ^b^	72.44 ± 3.50 ^c^
Hexanoic acid ^§^	ns	420 [31]	1870	Sweat	0.25 ± 0.05 ^a^	0.28 ± 0.03 ^a^	0.27 ± 0.01 ^a^	1.19 ± 0.14 ^b^	0.28 ± 0.01 ^a^	0.23 ± 0.01 ^a^	0.25 ± 0.03 ^a^
Nonanoic acid	***		2149	Must, fat	3.68 ± 0.77 ^a^	42.59 ± 4.68 ^b^	nd	30.16 ± 1.03 ^c^	nd	42.13 ± 0.03 ^b^	43.17 ± 2.08 ^b^
Octanoic acid ^§^	ns	0.5 [31]	2066	Cheese, sweat	2.66 ± 0.75 ^ab^	3.32 ± 0.90 ^a^	3.00 ± 0.27 ^a^	2.40 ± 0.18 ^b^	2.96 ± 0.17 ^ab^	3.43 ± 0.29 ^a^	3.37 ± 0.29 ^a^
Decanoic acid ^§^	ns	1 [28]	2269	Rancid, fat	0.64 ± 0.20 ^a^	0.99 ± 0.39 ^a^	0.76 ± 0.02 ^a^	0.65 ± 0.09 ^a^	0.99 ± 0.05 ^a^	2.14 ± 0.26 ^b^	2.17 ± 0.16 ^b^
*Alcohols*											
1-Butanol	***		1132		nd	2.38 ± 0.09 ^a^	48.63 ± 2.22 **^b^**	3.21 ± 0.07 ^a^	3.81 ± 0.05 ^a^	3.37 ± 0.26 ^a^	4.23 ± 0.20 ^a^
1-Dodecanol	**		1970		15.00 ± 2.73 ^ab^	10.94 ± 4.39 ^b^	17.24 ± 0.02 ^a^	12.19 ± 0.91 ^ab^	19.33 ± 1.39 ^a^	16.50 ± 0.87 ^ab^	16.50 ± 0.09 ^ab^
1-Hexanol ^§^	*	0.11 [32]	1351	Resin, flower, green	0.27 ± 0.01 ^a^	0.26 ± 0.01 ^ab^	0.24 ± 0.00 ^a^	0.23 ± 0.00 ^a^	0.25 ± 0.00 ^a^	0.23 ± 0.00 ^a^	0.19 ± 0.01 ^b^
1-Heptanol	***		1142		1.59 ± 0.69 ^a^	1.84 ± 0.09 ^a^	2.23 ± 1.75 ^ab^	4.45 ± 0.08 ^b^	nd	2.99 ± 0.15 ^a^	3.30 ± 0.29 ^ab^
1-Hexanol, 2- ethyl	***		1407	Citrus, rose	14.95 ± 0.77 ^a^	9.83 ± 0.55 ^b^	14.68 ± 1.30 ^a^	16.00 ± 3.36 ^a^	10.23 ± 0.74 ^b^	9.84 ± 0.20 ^b^	8.25 ± 0.40 ^b^
1-Octanol	***	120 [33]	1554	Grass	24.35 ± 0.85 ^a^	19.05 ± 1.96 ^b^	23.21 ± 2.05 ^ab^	15.77 ± 3.09 ^b^	16.61 ± 0.54 ^b^	20.42 ± 0.26 ^b^	27.60 ± 1.67 ^c^
1-Decanol	***		1780		10.70 ± 1.85 ^a^	18.83 ± 3.69 ^b^	19.80 ± 4.78 ^b^	13.47 ± 0.64 ^a^	19.89 ± 1.44 ^b^	30.88 ± 0.67 ^c^	35.25 ± 0.75 ^c^
2-Phenyl ethanol ^§^	***	14 [34]	1895	Honey, spicy, rose petal	0.73 ± 0.11 ^a^	0.99 ± 0.07 ^b^	0.89 ± 0.12 ^b^	1.23 ± 0.08 ^c^	0.95 ± 0.00 ^b^	1.20 ± 0.00 ^bc^	1.00 ± 0.06 ^b^
Isoamyl alcohol ^§^	***	30 [31]	1210	Spirit, alcoholic	4.43 ± 0.40 ^a^	5.07 ± 0.13 ^ab^	3.56 ± 0.40 ^c^	5.35 ± 0.12 ^b^	4.21 ± 0.03 ^ac^	5.06 ± 0.04 ^b^	4.79 ± 0.31 ^ab^
*Aldehydes*											
Furfural	***	14,100 [35]	1475	Almond, wood, caramel	39.04 ± 6.17 ^a^	9.62 ± 1.93 ^b^	28.35 ± 19.76 ^a^	11.45 ± 0.37 ^b^	4.40 ± 1.14 ^b^	nd	nd
Benzaldehyde	***	5 [36]	1506	Almond, sugar	nd	nd	nd	nd	nd	49.65 ± 4.55 ^a^	45.08 ± 1.53 ^b^
*Esters*											
Ethyl acetate ^§^	**	7.50 [30]	890	Pineapple	0.96 ± 0.16 ^a^	1.24 ± 0.15 ^b^	1.04 ± 0.14 ^a^	1.36 ± 0.04 ^b^	1.17 ± 0.43 ^b^	1.26 ± 0.03 ^b^	1.23 ± 0.03 ^b^
Isoamyl acetate ^§^	***	12 [28]	1133	Banana, fruit	0.11 ± 0.01 ^a^	0.08 ± 0.00 ^a^	0.09 ± 0.00 ^a^	0.12 ± 0.01 ^a^	0.32 ± 0.01 ^a^	1.51 ± 0.00 ^b^	2.86 ± 0.23 ^c^
Ethyl hexanoate ^§^	***	5 [30]	1235	Green apple, peach	2.71 ± 0.21 ^a^	4.08 ± 0.14 ^b^	nd	3.33 ± 0.08 ^c^	3.61 ± 0.04 ^b^	3.24 ± 0.05 ^c^	3.40 ± 0.06 ^b^
Ethyl-decanoate ^§^	***		1641	Grape, flower	1.33 ± 0.06 ^a^	1.52 ± 0.25 ^ab^	1.38 ± 0.02 ^a^	0.17 ± 0.04 ^c^	1.86 ± 0.20 ^b^	0.57 ± 0.03 ^d^	4.90 ± 0.12 ^e^
Ethyl lactate ^§^	***	150 [37]	1340	Milk, soap, butter, fruits	1.32 ± 0.27 ^a^	1.75 ± 0.14 ^b^	1.52 ± 0.09 ^b^	1.36 ± 0.31 ^a^	1.58 ± 0.46 ^b^	1.71 ± 0.82 ^b^	2.04 ± 0.18 ^c^
Ethyl nonanoate	***		1530		nd	nd	3.42 ± 0.90 ^a^	4.93 ± 1.20 ^a^	nd	11.93 ± 3.42 ^b^	12.48 ± 1.60 ^b^
Ethyl isovalerate	***		1038	Ripe fruits, pineapple, lemon, anice, flower	51.96 ± 4.78 ^a^	73.47 ± 5.54 ^b^	70.33 ± 6.79 ^b^	47.73 ± 0.67 ^a^	23.45 ± 3.75 ^c^	16.52 ± 0.42 ^c^	5.50 ± 0.60 ^d^
Hexyl acetate	***	115 [38]	1270	Coumarin, sweet	2.01 ± 0.51 ^a^	0.00 ± 0.00 ^b^	1.75 ± 0.22 ^a^	8.51 ± 0.10 ^c^	69.05 ± 5.75 ^d^	310.60 ± 6.58 ^e^	746.40 ± 0.72 ^f^
Phenylethyl acetate ^§^	***	250 [30]	1802	Rose, honey, tobacco	10.99 ± 0.48 ^a^	9.86 ± 1.21 ^a^	11.40 ± 0.39 ^a^	25.19 ± 1.56 ^b^	67.48 ± 0.73 ^c^	268.11 ± 13.19 ^d^	426.37 ± 29.65 ^e^
Ethyl pentanoate	***		1135		13.36 ± 0.72 ^a^	11.66 ± 0.70 ^a^	10.99 ± 0.70 ^a^	41.44 ± 2.19 ^b^	7.94 ± 0.95 ^c^	5.25 ± 0.16 ^c^	3.97 ± 1.40 ^c^
Ethyl dodecanoate	***		1853	Flower, fruits	nd	5.39 ± 2.25 ^a^	4.57 ± 3.49 ^a^	9.01 ± 1.56 ^b^	20.02 ± 1.57 ^c^	93.54 ± 11.28 ^d^	134.00 ± 15.74 ^e^
Diethyl malate	***		2029	Brown sugar, sweet	50.89 ± 0.73 ^a^	67.19 ± 8.30 ^b^	73.64 ± 28.59 ^c^	28.08 ± 9.29 ^d^	22.24 ± 0.93 ^d^	nd	nd
Ethyl butanoate	***		1032	Flower, fruits	86.43 ± 5.39 ^a^	119.61 ± 3.61 ^b^	123.45 ± 16.97 ^b^	96.67 ± 0.83 ^a^	97.52 ± 1.92 ^a^	125.30 ± 4.91 ^b^	163.49 ± 8.55 ^c^
Dimethyl succinate ^§^	***		1890		1.81 ± 0.27 ^a^	2.35 ± 0.17 ^b^	2.21 ± 0.29 ^ab^	1.69 ± 0.06 ^ac^	1.24 ± 0.02 ^c^	0.45 ± 0.02 ^d^	0.13 ± 0.01 ^d^
Methyl salycilate	***		1754		14.20 ± 2.06 ^a^	40.23 ± 11.10 ^b^	34.75 ± 25.67 ^b^	6.67 ± 0.62 ^a^	13.67 ± 2.45 ^a^	162.40 ± 10.98 ^c^	38.20 ± 2.05 ^b^
Ethylphenyl acetate	***		1798		70.65 ± 10.03 ^a^	16.51 ± 3.21 ^bc^	17.16 ± 2.60 ^bc^	22.87 ± 1.10 ^c^	9.10 ± 0.36 ^d^	12.44 ± 0.22 ^bd^	8.45 ± 0.95 ^bc^
2,2,4-Trimethyl-1,3-pentanediol diisobutirate	ns		--	Flower, rose petal	20.91 ± 4.30 ^a^	19.16 ± 5.89 ^a^	23.05 ± 1.95 ^ab^	19.32 ± 1.50 ^a^	20.44 ± 3.01 ^a^	26.69 ± 0.76 ^a^	21.44 ± 0.54 ^a^
*Norisoprenoids*											
3-Oxo-β-ionone	***		1921	Spicy, tobacco	53.90 ±6.53	nd	nd	nd	nd	nd	nd
β-Damascenone	***	0.05 [39]	1801	Flower, exotic fruits	2.49 ± 0.68 ^a^	2.62 ± 0.69 ^a^	6.03 ± 0.76 ^a^	3.02 ± 0.11 ^a^	nd	5.83 ± 0.15 ^a^	29.63 ± 5.18 ^b^
*Monoterpenes*											
Linalool	***	15 [28]	1551	Flower, lavender	0.41 ± 0.27 ^a^	1.69 ± 0.67 ^b^	0.71 ± 0.24 ^a^	0.66 ± 0.00 ^a^	0.00 ± 0.00 ^a^	3.39 ± 0.43 ^c^	4.27 ± 0.02 ^c^

Year of production is reported in brackets. Legend: nd, not detected. Different lowercase letters mean significant differences (F-test, *p* < 0.05). #: LS, Level of Significance: ns, non-significant; *, *p* < 0.05; **, *p* < 0.01; ***, *p* < 0.001.

**Table 4:** Descriptive quantitative profile of Trebbiano di Lugana wines from different vintages (1–13 years of storage).

Descriptor	LS #	Years of Storage						
		13 (2005)	9 (2009)	7 (2011)	5 (2013)	3 (2015)	2 (2016)	1 (2017)
*Visual descriptors*								
Yellow intensity	**	8.2 ± 0.1 ^a^	6.0 ± 1.0 ^b^	6.6 ± 1.0 ^b^	5.6 ± 1.3 ^c^	4.4 ± 1.2 ^c^	4.4 ± 1.0 ^c^	4.8 ± 1.6 ^c^
Clarity	ns	7.6 ± 2.0 ^a^	8.6 ± 0.7 ^a^	8.0 ± 1.5 ^a^	8.9 ± 0.4 ^a^	8.0 ± 1.4 ^a^	8.6 ± 0.7 ^a^	7.8 ± 1.6 ^a^
*Olfactory descriptors*								
Intensity	*	7.3 ± 1.5 ^a^	5.0 ± 1.7 ^b^	6.7 ± 1.1 ^a^	5.0 ± 1.8 ^b^	4.9 ± 1.0 ^a^	6.1 ± 1.7 ^a^	6.7 ± 1.2 ^a^
Fruity	**	2.4 ± 1.9 ^a^	3.6 ± 1.5 ^b^	2.9 ± 1.7 ^ab^	4.1 ± 2.3 ^b^	4.7 ± 1.7 ^c^	5.7 ± 1.5 ^c^	6.0 ± 1.5 ^c^
Boxwood/grapefruit/passion fruit	**	1.9 ± 1.2 ^a^	3.2 ± 2.0 ^a^	2.6 ± 1.3 ^a^	4.2 ± 2.1 ^b^	5.2 ± 1.2 ^b^	5.1 ± 2.0 ^b^	6.9 ± 0.8 ^b^
Floral	**	1.9 ± 1.3 ^a^	4.1 ± 2.0 ^b^	2.4 ± 1.9 ^a^	3.2 ± 1.3 ^b^	4.0 ± 1.9 ^b^	5.6 ± 1.4 ^c^	5.4 ± 1.6 ^c^
Oxidized/marsala	**	8.2 ± 0.7 ^a^	2.9 ± 1.0 ^ab^	6.3 ± 1.1 ^c^	3.0 ± 1.7 ^b^	1.6 ± 0.7 ^d^	1.7 ± 1.1 ^d^	1.3 ± 0.7 ^d^
Honey	**	6.3 ± 1.7 ^a^	2.8 ± 1.7 ^b^	4.7 ± 1.2 ^c^	2.7 ± 1.4 ^b^	2.5 ± 1.4 ^b^	2.7 ± 1.6 ^b^	2.0 ± 1.0 ^b^
*Taste-tactile descriptors*								
Body	ns	2.9 ± 0.9 ^a^	4.2 ± 2.1 ^a^	4.7 ± 1.3 ^a^	3.8 ± 1.8 ^a^	4.2 ± 1.7 ^a^	4.0 ± 1.6 ^a^	4.3 ± 1.1 ^a^
Acidity	*	3.0 ± 1.6 ^a^	5.3 ± 1.9 ^b^	5.0 ± 2.4 ^b^	4.4 ± 1.6 ^b^	5.1 ± 1.6 ^ab^	4.3 ± 2.0 ^ab^	5.2 ± 1.6 ^b^
Alcohol/heat	ns	4.0 ± 2.2 ^a^	5.3 ± 1.6 ^a^	5.3 ± 1.0 ^a^	3.4 ± 1.3 ^a^	3.8 ± 1.9 ^a^	5.0 ± 1.2 ^a^	4.8 ± 1.5 ^a^
Savory/salty	ns	3.8 ± 1.9 ^a^	4.4 ± 1.8 ^a^	4.7 ± 2.2 ^a^	3.9 ± 1.6 ^a^	4.1 ± 1.5 ^a^	4.7 ± 1.3 ^a^	4.8 ± 1.5 ^a^
Persistence	ns	5.0 ± 1.7 ^a^	6.3 ± 1.1 ^a^	6.2 ± 1.8 ^a^	5.8 ± 2.2 ^a^	5.9 ± 1.7 ^a^	6.4 ± 1.5 ^a^	6.4 ± 1.5 ^a^
Bitter	ns	3.1 ± 2.3 ^a^	2.6 ± 1.1 ^a^	2.7 ± 1.1 ^a^	3.0 ± 1.8 ^a^	1.9 ± 0.4 ^a^	2.6 ± 1.5 ^a^	2.3 ± 1.0 ^a^
*Aftertaste descriptors*								
Intensity	ns	5.1 ± 1.7 ^a^	6.0 ± 1.4 ^a^	5.8 ± 1.7 ^a^	5.2 ± 1.0 ^a^	5.3 ± 0.9 ^a^	6.3 ± 1.1 ^a^	6.1 ± 0.9 ^a^
Persistence	ns	5.6 ± 1.9 ^a^	6.3 ± 1.1 ^a^	6.2 ± 1.1 ^a^	5.8 ± 1.6 ^a^	5.3 ± 0.9 ^a^	7.1 ± 1.2 ^a^	6.3 ± 1.2 ^a^
Fruity	**	2.0 ± 1.0 ^a^	4.7 ± 1.6 ^bc^	3.3 ± 1.9 ^ab^	4.8 ± 1.7 ^cd^	5.7 ± 1.2 ^cd^	5.7 ± 1.7 ^d^	5.6 ± 1.7 ^d^
Peach	*	1.8 ± 1.3 ^a^	3.4 ± 1.5 ^ab^	2.9 ± 1.1 ^ab^	3.8 ± 1.3 ^b^	4.2 ± 1.7 ^b^	4.3 ± 1.3 ^b^	4.1 ± 1.8 ^b^
Marsala/honey/oxidized	**	8.1 ± 1.1 ^a^	2.8 ± 1.6 ^b^	6.3 ± 1.2 ^c^	2.7 ± 1.7 ^bd^	1.8 ± 1.0 ^bd^	1.9 ± 1.4 ^bd^	1.2 ± 0.4 ^d^
Green	ns	2.6 ± 1.4 ^a^	4.0 ± 1.7 ^a^	3.1 ± 1.6 ^a^	3.7 ± 1.8 ^a^	2.9 ± 1.3 ^a^	2.2 ± 1.5 ^a^	3.2 ± 1.4 ^a^

Year of production is reported in brackets. Different letters mean significant differences (F-test, *p* < 0.05). #: LS, Level of Significance: ns, non-significant; *, *p* < 0.05; **, *p* < 0.001.

## Supplementary Materials

The following are available online at https://www.mdpi.com/2304-8158/9/7/956/s1, Table S1: Pearson Correlation among the chemical parameters investigated and the storage time (1–13 years). The critical value is 0.755 (*n* = 5, *α* = 0.05). In bold are the significant correlation coefficients. Table S2: Pearson Correlation among the aroma compounds investigated and the storage time (1–13 years). The critical value is 0.755 (*n* = 5, *α* = 0.05). Figure S1: Relation between PC1 values and storage time (years).
