# Supplementary material for: Chemical Characterization and Volatile Profile of Trebbiano di Lugana Wine: A Case Study
Source: Foods. 2020 Jul 18;9(7):956. doi: 10.3390/foods9070956 (PMC7404698; doi:10.3390/foods9070956)
Supplement: Supplementary file 1 [file foods-09-00956-s001.pdf]

Table S1. Pearson Correlation among the chemical parameters investigated and the storage time (1–13 years). The critical value is 0.755 (df = 5, α = 0.05). In bold are the significant correlation coefficients.

**Parameters**	**Storage Time**	**L***	**a***	**b***	**Chrome**	**Hue**	**DO 420 %**	**DO 420 nm**	**GSH**	**TP**	**PP**	**Ratio TP/PP**	**OD 280 nm**	**OD 320 nm**	**Iron**	**Copper**
Storage time	1.00															
L*	−0.96	1.00														
a*	0.23	−0.44	1.00													
b*	**0.98**	−0.99	0.32	1.00												
Chroma	**0.98**	−0.99	0.31	**1.00**	1.00											
Hue	−0.95	0.97	−0.43	−0.97	−0.96	1.00										
OD 420 %	−0.97	**0.96**	−0.34	−0.97	−0.97	**0.99**	1.00									
OD 420 nm	**0.97**	−1.00	0.35	**1.00**	**1.00**	−0.97	−0.97	1.00								
GSH	−0.63	0.61	−0.53	−0.58	−0.57	0.65	0.67	−0.58	1.00							
TP	0.36	−0.33	−0.43	0.42	0.42	−0.28	−0.31	0.39	0.30	1.00						
PP	0.00	0.10	−0.53	−0.03	−0.02	0.17	0.09	−0.06	0.16	0.67	1.00					
Ratio TP/PP	−0.06	−0.05	0.58	−0.03	−0.04	−0.18	−0.10	−0.01	−0.15	−0.59	−0.89	1.00				
OD 280 nm	0.60	−0.49	−0.45	0.59	0.59	−0.45	−0.51	0.56	0.00	**0.90**	0.70	−0.68	1.00			
OD 320 nm	−0.13	0.36	−0.48	−0.31	−0.30	0.32	0.21	−0.32	−0.17	−0.09	0.57	−0.50	0.17	1.00		
Iron	0.74	−0.77	0.32	0.74	0.74	−0.66	−0.65	**0.76**	−0.20	0.25	−0.15	−0.01	0.40	−0.34	1.00	
Copper	0.28	−0.10	−0.52	0.17	0.17	−0.09	−0.15	0.16	0.33	0.25	0.02	−0.14	0.41	0.15	0.51	1.00

Legend: OD, optical density; GSH, glutathione; TP, total phenols; PP, polymeric phenols.

Table S2. Pearson Correlation among the aroma compounds investigated and the storage time (1–13 years). The critical value is 0.755 (df = 5, α = 0.05).

	ST	**1**	**2**	**3**	**4**	**5**	**6**	**7**	**8**	**9**	**10**	**11**	**12**	**13**	**14**	**15**	**16**	**17**	**18**	**19**	**20**	**21**	**22**	**23**	**24**	**25**	**26**	**27**	**28**	**29**	**30**	**31**	**32**	**33**	**34**	**35**	**36**
ST	1.00																																				
1	−0.64	1.00																																			
2	−0.06	−0.24	1.00																																		
3	−0.43	0.55	−0.71	1.00																																	
4	−0.40	0.42	0.13	0.52	1.00																																
5	−0.72	0.72	−0.39	**0.78**	0.65	1.00																															
6	0.13	0.14	−0.16	−0.02	−0.49	−0.28	1.00																														
7	−0.20	−0.04	0.49	0.03	**0.82**	0.30	−0.77	1.00																													
8	0.61	−0.86	0.05	−0.62	−0.79	−0.73	0.14	−0.40	1.00																												
9	−0.26	0.35	0.63	−0.13	0.60	0.25	−0.04	0.57	−0.50	1.00																											
10	0.57	−0.54	0.57	−0.87	−0.53	−0.78	0.38	−0.20	0.64	0.23	1.00																										
11	0.07	0.65	−0.56	0.37	0.06	0.42	0.23	−0.31	−0.38	0.09	−0.21	1.00																									
12	−0.79	**0.85**	−0.41	**0.82**	0.57	**0.96**	−0.07	0.12	−0.80	0.21	−0.79	0.44	1.00																								
13	−0.66	0.16	0.57	0.11	0.65	0.40	−0.27	0.68	−0.44	0.65	−0.15	−0.49	0.36	1.00																							
14	−0.44	0.29	−0.49	0.20	−0.52	0.29	0.28	−0.66	0.15	−0.48	−0.23	0.23	0.37	−0.25	1.00																						
15	**0.87**	−0.47	−0.05	−0.55	−0.66	−0.71	0.44	−0.51	0.67	−0.19	**0.76**	0.25	−0.72	−0.72	−0.09	1.00																					
16	−0.67	0.69	−0.30	0.67	0.63	**0.98**	−0.26	0.33	−0.67	0.38	−0.63	0.45	**0.90**	0.42	0.26	−0.61	1.00																				
17	−0.63	0.20	0.58	0.13	0.73	0.35	−0.44	**0.78**	−0.56	0.56	−0.30	−0.50	0.34	**0.93**	−0.38	−0.81	0.32	1.00																			
18	−0.59	0.95	−0.34	0.75	0.58	**0.78**	0.13	0.05	−0.92	0.33	−0.66	0.60	**0.89**	0.21	0.13	−0.53	0.72	0.26	1.00																		
19	**0.77**	−0.54	0.14	−0.33	−0.29	−0.77	0.48	−0.24	0.41	−0.10	0.57	−0.20	−0.71	−0.33	−0.54	0.65	−0.77	−0.31	−0.41	1.00																	
20	−0.69	**0.88**	−0.28	0.60	0.57	**0.92**	−0.26	0.24	−0.80	0.34	−0.67	0.59	**0.91**	0.26	0.29	−0.60	**0.91**	0.28	**0.84**	−0.81	1.00																
21	−0.24	−0.02	0.12	0.19	0.60	0.33	−0.95	**0.78**	−0.32	0.01	−0.55	−0.27	0.18	0.34	−0.33	−0.61	0.25	0.57	0.04	−0.43	0.30	1.00															
22	−0.67	**0.90**	−0.27	0.58	0.55	**0.88**	−0.25	0.21	−0.81	0.32	−0.67	0.61	**0.89**	0.22	0.29	−0.58	**0.86**	0.27	**0.85**	−0.79	**1.00**	0.30	1.00														
23	−0.61	**0.82**	−0.45	**0.86**	0.64	**0.82**	−0.17	0.18	−0.89	0.08	−0.90	0.43	0.90	0.21	0.13	−0.70	0.70	0.34	**0.91**	−0.51	**0.82**	0.36	0.82	1.00													

24	−0.73	0.75	−0.02	0.48	0.63	**0.87**	−0.10	0.33	−0.72	0.63	−0.42	0.40	**0.84**	0.57	0.18	−0.57	**0.93**	0.44	0.74	−0.70	**0.87**	0.10	**0.84**	0.61	1.00												
25	0.18	−0.41	**0.97**	−0.81	0.00	−0.57	−0.09	0.41	0.23	0.54	0.72	−0.55	−0.61	0.40	−0.57	0.17	−0.47	0.40	−0.50	0.34	−0.47	0.03	−0.46	−0.62	−0.21	1.00											
26	−0.28	**0.78**	−0.42	0.40	0.13	0.42	−0.05	−0.19	−0.61	−0.11	−0.55	0.68	0.53	−0.34	0.28	−0.22	0.32	−0.13	0.68	−0.40	0.66	0.16	0.72	0.71	0.27	−0.51	1.00										
27	**0.77**	−0.65	0.18	−0.44	−0.43	−0.88	0.41	−0.28	0.54	−0.24	0.60	−0.33	−0.81	−0.37	−0.46	0.65	−0.89	−0.32	−0.56	**0.97**	−0.90	−0.38	−0.88	−0.60	−0.83	0.38	−0.42	1.00									
28	−0.40	0.17	−0.34	0.65	0.49	0.71	−0.08	0.25	−0.26	0.19	−0.43	0.08	0.60	0.46	0.14	−0.43	0.75	0.23	0.35	−0.36	0.41	0.06	0.33	0.36	0.63	−0.43	−0.28	−0.49	1.00								
29	**0.83**	−0.53	0.00	−0.55	−0.40	−0.50	−0.11	−0.09	0.62	−0.11	0.59	0.23	−0.66	−0.60	−0.21	**0.83**	−0.37	−0.64	−0.59	0.33	−0.39	−0.14	−0.39	−0.64	−0.43	0.19	−0.21	0.35	−0.30	1.00							
30	−0.73	**0.84**	−0.28	0.62	0.58	**0.95**	−0.28	0.26	−0.77	0.33	−0.69	0.53	**0.92**	0.32	0.32	−0.64	**0.94**	0.31	**0.81**	−0.84	**0.99**	0.31	**0.98**	**0.80**	**0.89**	−0.47	0.60	−0.93	0.48	−0.42	1.00						
31	−0.74	**0.83**	−0.27	0.64	0.61	**0.96**	−0.28	0.28	−0.78	0.35	−0.70	0.51	**0.94**	0.36	0.31	−0.66	**0.95**	0.34	**0.81**	−0.83	**0.99**	0.31	**0.97**	**0.81**	**0.91**	−0.47	0.55	−0.93	0.53	−0.45	**1.00**	1.00					
32	−0.37	0.25	−0.42	0.50	0.11	0.61	0.25	−0.15	−0.08	0.12	−0.21	0.28	0.56	0.22	0.48	−0.16	0.68	−0.09	0.31	−0.38	0.39	−0.33	0.32	0.20	0.62	−0.48	−0.21	−0.49	**0.88**	−0.18	0.45	0.49	1.00				
33	**0.79**	−0.46	−0.06	−0.26	−0.45	−0.76	0.57	−0.46	0.44	−0.29	0.50	−0.05	−0.66	−0.54	−0.35	0.71	−0.78	−0.50	−0.36	**0.96**	−0.76	−0.52	−0.74	−0.45	−0.75	0.15	−0.23	**0.95**	−0.44	0.35	−0.81	−0.81	−0.37	1.00			
34	0.74	−0.62	0.55	−0.60	0.03	−0.69	−0.11	0.35	0.33	0.27	0.64	−0.31	−0.78	−0.05	−0.86	0.51	−0.62	0.02	−0.55	0.73	−0.65	0.06	−0.64	−0.59	−0.53	0.71	−0.46	0.72	−0.41	0.56	−0.68	−0.68	−0.57	0.58	1.00		
35	−0.50	**0.95**	−0.19	0.45	0.46	0.69	−0.05	0.09	−0.83	0.40	−0.49	0.74	0.75	0.07	0.16	−0.36	0.68	0.15	**0.88**	−0.55	**0.90**	0.11	**0.93**	**0.76**	0.73	−0.34	**0.83**	−0.66	0.08	−0.30	**0.85**	**0.83**	0.13	−0.48	−0.46	1.00	
36	−0.57	**0.78**	−0.28	0.74	**0.79**	**0.93**	−0.24	0.40	−0.86	0.46	−0.68	0.53	**0.90**	0.38	0.00	−0.60	**0.93**	0.38	**0.87**	−0.59	**0.91**	0.31	**0.89**	**0.84**	**0.88**	−0.44	0.47	−0.75	0.61	−0.41	**0.91**	**0.93**	0.47	−0.61	−0.46	**0.80**	1.00

Legend: ST, Storage time; 1, Acetic acid; 2, Hexanoic acid; 3, Octanoic acid; 4, Nonanoic acid; 5, Decanoic acid; 6, 1-Butanol; 7, Isoamyl alcohol; 8, 1-Hexanol; 9, 1-Heptanol: 10, 1-Hexanol, 2- ethyl; 11, 1-Octanol; 12, 1-Decanol; 13, 2-Phenyl ethanol; 14, 1-Dodecanol; 15, Furfural; 16, Benzaldehyde; 17, Ethyl acetate; 18, Ethyl butanoate, 19, Ethyl isovalerate; 20, Isoamyl acetate; 21, Ethyl hexanoate; 22; Hexyl acetate, 23, Ethyl lactate; 24, Ethyl nonanoate; 25, Ethyl pentanoate; 26, Ethyl decanoate; 27, Dimethyl succinate; 28, Methyl salycilate; 29, Ethylphenyl acetate; 30, Phenylethyl acetate; 31, Ethyl dodecanoate; 32, 2,2,4-Trimethyl-1,3-pentanediol disobutirate; 33, Diethyl malate; 34, 3-Oxo-β-ionone; 35, β-Damascenone; 36, Linalool.

Figure S1. Relation between PC1 values and storage time (years).

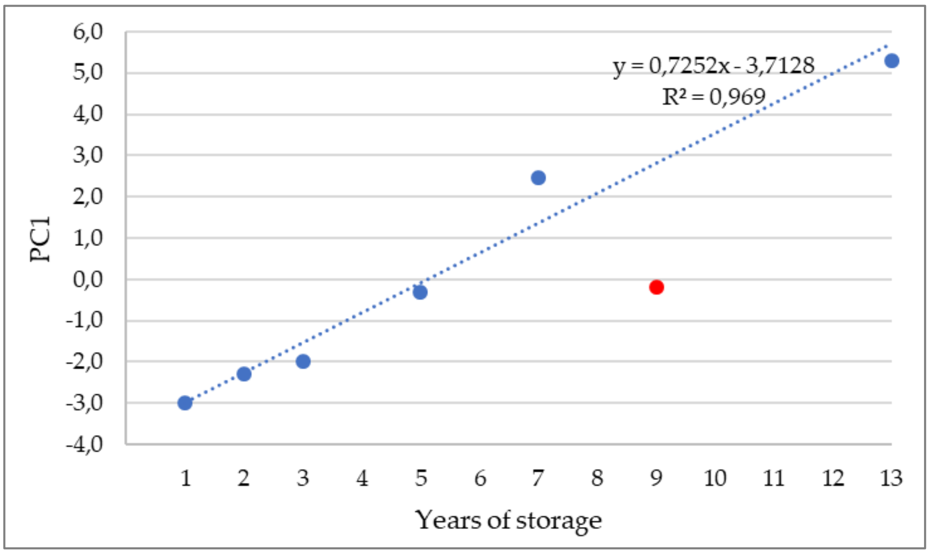

The wine produced in 2009 was excluded from the linear modelling years of storage-PC1. Legend: Blue markers represented the wine samples produced in 2005, 2011, 2013, 2015, 2016 and 2017; red marker represented the wine sample produced in 2009.
